# Electrochemical Sensor of Double-Thiol Linked PProDOT@Si Composite for Simultaneous Detection of Cd(II), Pb(II), and Hg(II)

**DOI:** 10.3390/polym11050815

**Published:** 2019-05-07

**Authors:** Mihray Abdulla, Ahmat Ali, Ruxangul Jamal, Tursunnisahan Bakri, Wei Wu, Tursun Abdiryim

**Affiliations:** 1 Key Laboratory of Petroleum and Gas Fine Chemicals, Educational Ministry of China, School of Chemistry and Chemical Engineering, Xinjiang University, Urumqi 830046, China; mihrayabdulla@163.com (M.A.); ahmatjanchem@126.com (A.A.); tursunnisabakri@sina.com (T.B.); wu10001300@163.com (W.W.); 2 Key Laboratory of Functional Polymers, Xinjiang University, Urumqi 830046, China

**Keywords:** thiol(–SH), poly(3,4-proplenedioxythiophene), porous silicon spheres, electrochemical sensor, heavy metal ions, simultaneous detection

## Abstract

Heavy metal ions in water, cosmetics, and arable land have become a world-wide issue as they cause a variety of diseases and even death to humans and animals when a certain level is exceeded. Therefore, it is necessary to development a new kind of sensor material for the determination of heavy metal ions. In this paper, we present an electrochemical sensor based on composite material (thiol(–SH) grafted poly(3,4-proplenedioxythiophene) (PProDOT(MeSH)_2_)/ porous silicon spheres (Si) composite, denoted as PProDOT(MeSH)_2_@Si) from the incorporation of thiol(–SH) grafted poly(3,4-proplenedioxythiophene) (PProDOT(MeSH)_2_) with porous silicon spheres (Si) for the electrochemical detection of heavy metal ions (Cd(II), Pb(II), and Hg(II)). The PProDOT(MeSH)_2_@Si composite was synthesized via a chemical oxidative polymerization method. The structure and morphology of PProDOT(MeSH)_2_@Si composite were characterized by Fourier transform infrared (FT-IR), Ultraviolet–visible spectroscopy (UV–Vis), X-ray diffraction (XRD), scanning electron microscope (SEM), Transmission electron microscope (TEM), and Brunauer−Emmett−Teller (BET). Furthermore, the electrochemical performance of PProDOT(MeSH)_2_@Si was evaluated by detecting of Cd(II), Pb(II), and Hg(II) ions using the differential pulse voltammetry (DPV) method. The relationship between structural properties and the electrochemical performance was systematically studied. The results showed that the entry of two thiol-based chains to the monomer unit resulted in an increase in electrochemical sensitivity in PProDOT(MeSH)_2,_ which was related to the interaction between thiol group(-SH) and heavy metal ions. And, the combination of PProDOT(MeSH)_2_ with Si could improve the electrocatalytic efficiency of the electrode material. The PProDOT(MeSH)_2_@Si/GCE exhibited high selectivity and sensitivity in the rage of 0.04 to 2.8, 0.024 to 2.8, and 0.16 to 3.2 μM with the detection limit of 0.00575, 0.0027, and 0.0017 µM toward Cd(II), Pb(II), and Hg(II), respectively. The interference studies demonstrated that the PProDOT(MeSH)_2_@Si/GCE possessed a low mutual interference and high selectivity for simultaneous detection of Cd(II), Pb(II), and Hg(II) ions.

## 1. Introduction

With the development of the economy and industry, environmental pollution has become a serious global issue, particularly, heavy metal pollution [1]. Heavy metal ions, such as mercury, cadmium, lead, chromium, and metalloids, are a factor for teratogenicity and carcinogenicity of normal cells [2]. Therefore, it is necessary to develop a rapid, low-cost, high sensitivity, and selective analytical method for the detection of heavy metal ions.

In recent years, electrochemical analysis has attracted great attention with its effective means to solve the environmental issues related to industrial processes. Electrochemical methods, such as square wave anodic stripping voltammetry (SWASV), differential pulse stripping voltammetry (DPSV), square wave stripping voltammetry (SWSV), adsorptive stripping differential pulse voltammetry (ASDPV), and differential pulse voltammetry (DPV), have been widely used for the determination of heavy metal ions due to their simple, rapid, highly sensitive characteristics [3,4]. The performance of electrochemical sensors is closely related to the electrode materials. Hence, preparation of electrode materials with high sensitivity, high selectivity, and weak mutual interference is highly desirable. Generally, these types of electrode materials are synthesized using metallic nanomaterials [5], metal oxides-based nanomaterials [6,7], graphene-based nanocomposites [8,9], polymers [10], and polymers-based nanocomposities [11,12]. Among them, conducting polymers-based nanomaterials are the kind of novel materials, in which, polymers can be blended with an inorganic substrate to form composite materials, and their mechanical, electrical, and other properties can be greatly improved. Thus, conducting polymers-based nanomaterial can be used as electrochemical sensors to detect various small biological molecules, heavy metal ions, and other species [13].

Poly(3,4-ethylenedioxythiophene) (PEDOT) and its derivatives are typical electrode sensor materials, due to their low oxidation potential, high thermal and chemical stability [14,15]. In addition, PEDOT and its derivatives also contain various special functional groups and donor atoms which can increase their coordination ability with metal ions [16] and improve the selectivity and sensitivity for electrochemical detection of heavy metals. The poly(3,4-proplenedioxythiophene) (PProDOT) is a conjugated polymer and is similar to PEDOT in terms of performance. The high electron-richness and excellent co-planarity, stable electroactive property of PProDOT is the reason it to became a useful building block in the synthesis of novel polymeric composites [17,18]. Therefore, PProDOT and its derivatives have great potential to be applied for the detection of heavy metal ions. However, there are few reports, so far, on its application in the sensor materials for electrochemical detection. It is generally considered that this type of conducting polymer with specific adsorption functional groups (such as –CN, –NH_2_, –SH, etc.) have the ability to form strong chemical bonds with the positively charged heavy metal ions (such as Hg^2+^, Cd^2+^, Pb^2+^, and Ag^+^) resulting from the interaction between conventional acid and base [19], especially the thiol(–SH) groups [9]. In our previous report, poly(EDOT-pyridine-EDOT) and poly(EDOT-pyridazine-EDOT) hollow nano-sphere materials [20] and Poly(EDOT-pyridine-EDOT)/graphitic carbon nitride composite [21] were successfully prepared and applied in the detection of Cd^2+^, Pb^2+^, and Cu^2+^, and we found that O, S, N atoms in PEDOT derivatives provide large active sites and displayed a strong interaction with heavy metal ions.

Porous silicon materials have been widely applied in medicine, water treatment, optical devices, energy, and chemical and biological sensors due to their high crystallinity, large specific surface area, optimal band structure, and good chemical and physical stability [22]. The structural design and stable coating layer (such as functional groups, biomolecules, and polymers) with high sensitivity for heavy metal ions on the porous silicon surface are considered as one of the most promising ways to enhance the chemical stability and sensitivity of porous silicon. Recently, porous silicon materials functionalized with functional groups or biomolecules have been synthesized and employed as adsorbents to remove heavy metal ions from waste water [23,24]. Zheng at al. designed and fabricated a thiol and amino groups grafted Si nanowires modified electrode to detect Cd(II) and Pb(II) ions [25]. However, the layer (functional groups or biomolecules) used to graft porous silicon has poor electrochemical stability and sensitivity [26]. Therefore, it should be noted that the incorporation of a conducting polymer with porous silicon material can be an effective way to overcome these drawbacks to improve the electrochemical sensing performance of the porous silicon material for the target ions. Thiol(–SH) grafted PEDOT type conducting polymer is preferred to combine with porous silicon material to enhance the electrochemical stability and sensitivity by the structural advantages, such as containing donor atoms (S and O) and thiol groups (–SH) which accelerate the coordination of ions.

In the present work, we synthesized a thiol(–SH) grafted monomer (double-thiol linked ProDOT), and this monomer was used to in-situ oxidative polymerization to prepare the double-thiol linked PProDOT/porous silicon spheres (PProDOT(MeSH)_2_@Si) composite in which the silicon spheres (Si) were prepared by magnesium thermal reduction of silica spheres. The composite was used as an electrode material for the electrochemical detection of heavy metal ions (Cd(II), Pb(II), and Hg(II)). The PProDOT(MeSH)_2_@Si composite was synthesized via a chemical oxidative polymerization method. The structure and morphology of PProDOT(MeSH)_2_@Si composite were characterized by Fourier transform infrared (FT-IR), Ultraviolet–visible spectroscopy (UV–Vis), X-ray diffraction (XRD), scanning electron microscope (SEM), Transmission electron microscope (TEM), and Brunauer−Emmett−Teller (BET). Furthermore, the electrochemical performance of PProDOT(MeSH)_2_@Si was evaluated by detecting of Cd(II), Pb(II), and Hg(II) ions using the differential pulse voltammetry (DPV) method. The relation between structural properties and the electrochemical performance was systematically studied to evaluate the potential application of PProDOT(MeSH)_2_@Si as a material for an electrochemical sensor for the detection of heavy metal ions (Cd(II), Pb(II), and Hg(II)).

## 2. Materials and Methods

### 2.1. Chemicals and Reagents

3,4-Di(methoxy)thiophene(DMOT), 2,2-Bis(bromomethyl)propane-1,3-diol, *p*-Toluenesulfonic acid (*p*-TSA, Aladdin, Shanghai, China), thioacetic acid s-potassium salt, Toluene, *N*,*N*-dimethylformamide (DMF), Tetrahydrofuran (THF), Anhydrous FeCl_3_, Tetraethyl orthosilicate (TEOS, 98%; Aladdin, Shanghai, China), ammonium, Etyltriethylammnonium bromide (CTAB), Magnesiumpowder (Mg, 99.9%; Aladdin, Shanghai, China).

### 2.2. Synthesis of Monomer

**Scheme 1:**
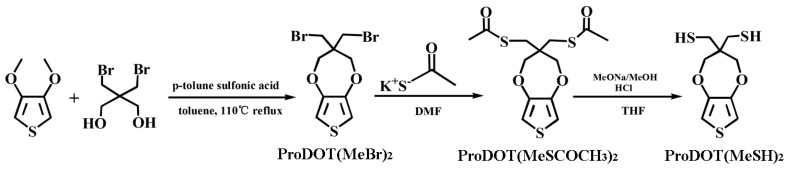
The synthesis route of double-thiol linked (3,4-proplenedioxythiophene) ProDOT(MeSH)_2_ monomer.

Scheme 1 illustrates the synthesis route of ProDOT(MeSH)_2_ monomer, and the detailed synthesis process of monomer are shown as follows:

#### 2.2.1. Synthesis of ProDOT(MeBr)_2_

**Figure 1:**
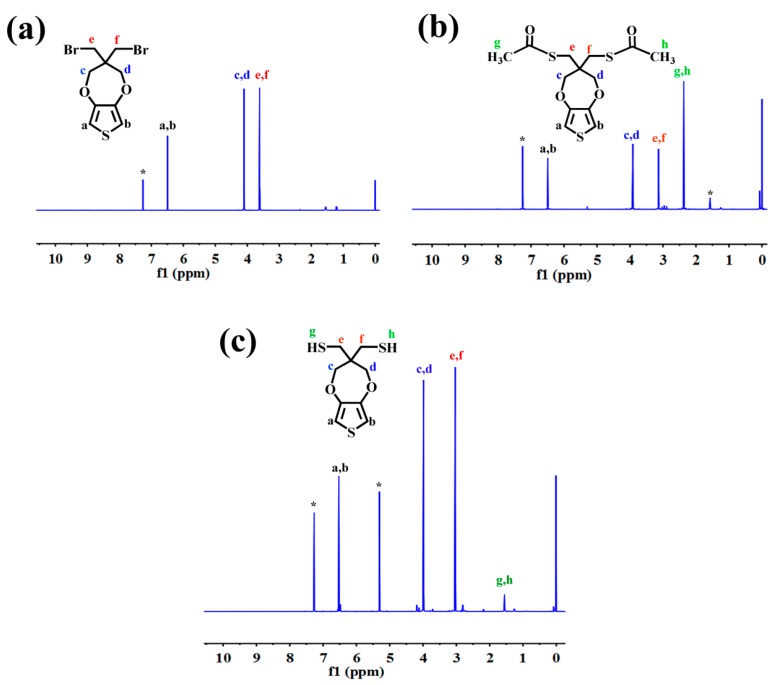
^1^H NMR spectra of ProDOT(MeSH)_2_ in CDCl_3._

ProDOT(MeBr)_2_ was synthesized based on a previous reports [27,28]. 3,4-dimethoxythiophene (3.03 g, 21 mmol), 2,2-bis(bromomethyl) propane-1,3-diol (11.0 g, 42 mmol), p-toluenesulfonic acid (0.4 g, 2.1 mmol) and 200 mL of toluene were placed in a 500 mL two-neck flask and magnetically stirred at 110 °C for 48 h under a nitrogen atmosphere. The reaction mixture was cooled to room temperature and washed with deionized water. Finally, the toluene was removed under vacuum, and the crude product was purified by column chromatography on silica gel with 3:2 hexanes/methylene chloride. A yield of 87% white crystalline solid was obtained and characterized by Nuclear Magnetic Resonance Spectroscopy ^1^H NMR (Figure 1a). ^1^H NMR: (400 MHz, CDCl_3_): δ [ppm] 6.49 (s, 2H), 4.10 (s, 4H), 3.61 (s, 4H) as in Figure 1a.

#### 2.2.2. Synthesis of ProDOT(MeSCOMe)_2_

ProDOT(MeBr)_2_ (1.5 g, 367 mmol) and thioacetic acid s-potassium salt (3.0 g, 9.4 mmol) were added to *N,N*-dimethylformamide (DMF, 12 mL) and refluxed for 15 h at 50 °C. After that, the reaction mixture was cooled to room temperature and extracted with dichloromethane (40 mL CH_2_Cl_2_) by replacing the water three times. Finally, the organic phase was dried with anhydrous magnesium sulfate [16,29]. Yield of 86% orange red oil were obtained after removing dichloromethane under reduced pressure. ^1^H NMR (400 MHz, CDCl_3_): δ [ppm] 6.49 (s, 2H), 3.90 (s, 4H), 3.14 (s, 4H), 2.38 (s, 6H) as in Figure 1b.

#### 2.2.3. Synthesis of ProDOT(MeSH)_2_

ProDOT(MeSH)_2_ was synthesized based on a previous report [16]. ProDOT(MeSCOMe)_2_ (1.0 g, 4.3 mmol) and sodium methoxide (1.2 M in methanol, 8.0 mL) were added to distilled THF (60 mL) and stirred at room temperature for 8 h. Finally, the reaction mixture was treated with 5.0 M HCl before evaporation of ProDOT(MeSH)_2_. The ^1^H NMR spectrum of the monomer is shown in Figure 1c: yield of 72% origin crystalline solid, ^1^H NMR (400 MHz, CDCl_3_): δ [ppm] 6.55 (s, 2H) δ, 3.97 (s, 4H), 3.03 (s, 4H), 1.57 (s, 2H) as in Figure 1c.

### 2.3. Preparation of Composite

**Scheme 2:**
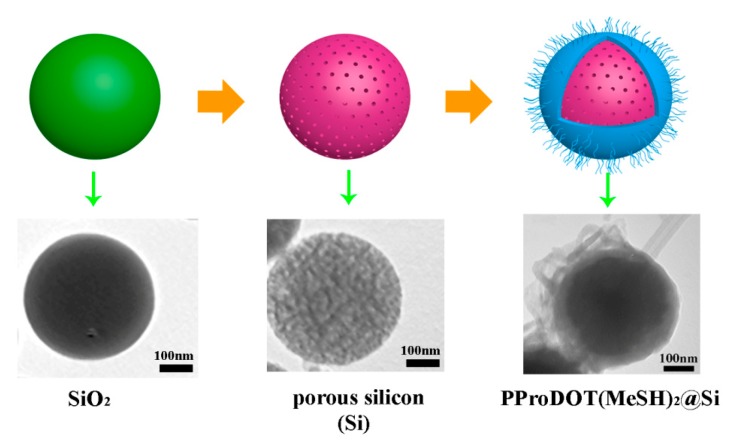
The synthesis route of PProDOT(MeSH)_2_@Si composite.

Scheme 2 illustrates the synthesis route of the PProDOT(MeSH)_2_@Si composite. The composite was prepared by three step: first step, synthesis of SiO_2_ spheres; second step, synthesis of SiO_2_ spheres; third step, fabrication of PProDOT(MeSH)_2_@Si composites.

#### 2.3.1. Synthesis of SiO_2_ Spheres

SiO_2_ spheres (average diameter 360 nm) were prepared by a modified Stöber method [30]. Ethanol (45.5 mL, containing 4.5 mL TEOS) was rapidly added into a mixture of ethanol (16.25 mL), H_2_O (24.75 mL), and ammonium (28%) (9 mL). Then, the mixture was allowed to react for 2 h.

#### 2.3.2. Synthesis of Porous Silicon Spheres (Si)

Porous silicon spheres (Si) were prepared base on previous reports [31,32]. SiO_2_ spheres and magnesium (Mg) powder were mixed in a mass ratio of 1:1 and placed on an alumina boat. The reduction reaction was realized by heat-treatment at 700 °C for 6 h under Nitrogen (N_2_) atmosphere. After the reaction, the mixture was cooled to room temperature, and 1.0 M hydrochloric acid (HCl) solution was added and stirred for 5 h to remove the generated magnesium oxide (MgO) and residual Mg. Finally, the yellow–brown powder was thoroughly washed by deionized water and absolute ethanol and dried at 60 °C for 12 h.

#### 2.3.3. Fabrication of PProDOT(MeSH)_2_@Si Composites

Fifteen milligram of porous silicon sphere (Si) and 80 mg CTAB were dispersed in 20 mL CHCl_3_. Then 10 mL of CHCl_3_ containing 20 mg of ProDOT(MeSH)_2_ monomer were dropped into the above suspension and stirred for 20 min. Finally, 100 mg FeCl_3_ was added to the mixture solution and stirred at room temperature for 24 h. The resultant products were centrifuged at 1700 rpm, thoroughly washed with deionized water and absolute ethanol, and dried at 60 °C for 24 h. The obtained composites were denoted as PProDOT(MeSH)_2_@Si. In addition, pure PProDOT and PProDOT(MeSH)_2_ were synthesized by the same approach without Si.

### 2.4. Structure Characterization

Fourier transform infrared (FTIR) spectra of the samples were recorded over BRUKER-QEUINOX-55 FTIR spectrometer using KBr pellets. UV–Vis spectra of the samples were recorded over UV–visible spectrophotometer (UV4802, Unico, Shanghai, China). Morphology and microstructure of the samples were characterized by a scanning electron microscope (SEM, SO8010, Japan). The elemental percentage of the sample was carried on X-ray diffraction (XRD), and the scan range (2è) was 10° to 80°.

### 2.5. Measurement of Electrocatalytic Activity

All electrochemical testing procedures were executed using electrochemical workstation CHI 660C (ChenHua Instruments Co, Shanghai, China) at room temperature. The three electrode system was Pt electrode, saturated calomel electrode (SCE) and PProDOT(MeSH)_2_@Si modified GCE (glassy carbon electrode; diameter = 4 mm) was used, respectively, as a used counter electrode, reference electrode, and working electrode. The working electrode was prepared by placing 5.0 μL of 1.0 mg/mL samples of the to be tested suspension on the electrode surface, finally dried at room temperature for 30 min. The differential pulse voltammetry (DPV) method was applied for the determination of Pb(II), Cd(II), and Hg(II), performed in 0.1 M acetic acid/sodium acetate buffer solution (ABS) with pH value 4.5 in the potential range from −1.4 to 0.4 V. Deposition potential: −1.4 V, deposition time: 220 s, amplitude: 0.05 V, increment potential: 0.002 V, pulse width: 0.05 s, pulse period: 0.1 s.

## 3. Results and Discussion

### 3.1. Structure Characterization

**Figure 2:**
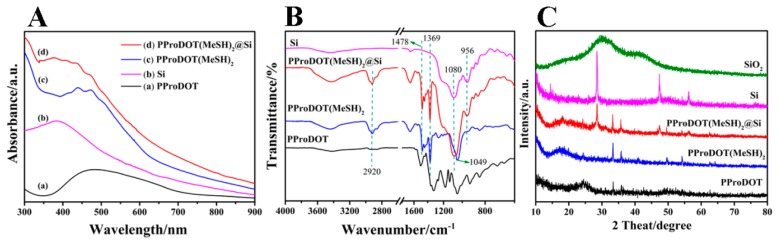
(**A**) UV–Vis spectra, (**B**) Fourier transform infrared (FT-IR) spectra, (**C**) X-ray diffraction (XRD) patterns of porous silicon spheres (Si), PProDOT, PProDOT(MeSH)_2_ and PProDOT(MeSH)_2_@Si composite.

Figure 2A shows the UV–Vis adsorption spectra of the porous silicon spheres (Si), PProDOT, PProDOT –(MeSH)_2_ and PProDOT(MeSH)_2_@Si composite. The absorption peak of porous silicon spheres (Si) appears at 380 nm, which is originated from the oxidation of silicon on porous silicon spheres. The PProDOT shows characteristic absorption peaks at ~456 and ~625 nm, while the absorption peaks of PProDOT(MeSH)_2_ appear at ~430 and ~485 nm, which are assigned to the π–π* transition of the thiophene ring [33]. In the case of poly PProDOT(MeSH)_2_@Si composite, there are similar absorption peaks to that of PProDOT(MeSH)_2_. Moreover, the absorption peak appears at 380 nm in PProDOT(MeSH)_2_@Si confirms that the presence of Si in PProDOT(MeSH)_2_@Si composite.

Figure 2B indicates the FTIR spectra of porous silicon spheres (Si), PProDOT, PProDOT(MeSH)_2_, and PProDOT(MeSH)_2_@Si composite. The characteristic vibration bands of porous silicon spheres (Si) appear at ~956 and ~1080 cm^−1^, which corresponds to the deformation vibrations of Si−O mode arising from the thin layer of SiO_2_ on surface of porous silicon spheres [19,34,35]. For PProDOT, the typical vibration bands locate at ~1492, ~1320, ~1176, ~1128, ~1049, ~930, ~844, and ~710 cm^−1^, while pure PProDOT(MeSH)_2_ displays vibration bands at ~1656, ~1478, ~1260, ~1049, ~842, and ~738 cm^−1^. Among them, the vibration bands at ~1656, ~1492, ~1478, and ~1320 cm^−1^ are assigned to C=C and C–C stretching mode of thiophene ring [36]. The bands at ~1260, ~1176, ~1128, ~1049, and ~1045 cm^−^^1^ are from the C–O–C bending vibration in the ethylenedioxy group. The vibration bands at ~930, ~840, and ~700 cm^−1^ are assigned to vibrations of the C–S–C bond stretching in thiophene ring [37]. In addition, several vibration bands for PProDOT(MeSH)_2_ are observed around 2800 to 3000 and 3200 to 3700 cm^−1^, respectively. The band at 2800 to 3000 cm^−1^ is attributed to the out-plane deformation of the polymer chain alkyl groups (–CH_2_, –CH_3_) [38]. The broad bands at 3200 to 3700 cm^−1^ are characteristic vibrations of –OH and S–H [16]. The FTIR spectrum of PProDOT(MeSH)_2_@Si composite shows nearly identical positions comparing with pure PProDOT(MeSH)_2_. Aside from the main characteristic bands of PProDOT(MeSH)_2_, the characteristic bands of Si (~956 and ~1080 cm^−1^) also appear in PProDOT(MeSH)_2_@Si composite.

**Figure 3:**
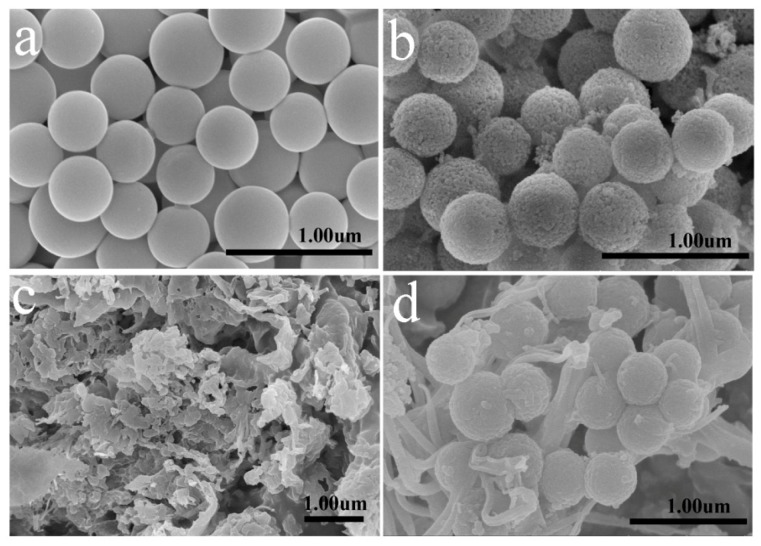
SEM images of (**a**) SiO_2_, (**b**) porous silicon sphere (Si), (**c**) PProDOT(MeSH)_2_, (**d**) PProDOT(MeSH)_2_@Si.

Figure 2C exhibits the XRD patterns of SiO_2_ spheres, porous silicon spheres (Si), PProDOT, PProDOT(MeSH)_2_, and PProDOT(MeSH)_2_@Si composite. As shown in Figure 3C, the SiO_2_ spheres exhibit only a broad diffraction peak at 2θ = 23°~38° indicating that the SiO_2_ spheres are amorphous. In the case of porous silicon spheres, diffraction peaks are located at 28.4°, 47.4°, 56.2°, which can be assigned to the (111), (220), and (311) planes of the crystalline phase of silicon spheres [32]. The characteristic diffraction peak of PProDOT is located at 2θ = 24°, while that of PProDOT(MeSH)_2_ is located at 2θ = 17.5°. In comparison with PProDOT, the diffraction peak of PProDOT(MeSH)_2_ at 2θ = 17.5° reflects reflexes the larger distance of polymer chains separated by two thiol-based chains entry to the monomer unit than that of PProDOT. Furthermore, the absence of diffraction peak at 2θ = 24° in PProDOT(MeSH)_2_ indicates the intermolecular π–π* stacking distance becomes larger than that of PProDOT, implying that the entry of two thiol-based chains to monomer unit results in an increase of amorphous structure in the polymer matrix [36]. In addition, the several diffraction peaks at 33.3°, 35.8°, 49°, 54° appear in pure PProDOT, PProDOT(MeSH)_2_, and PProDOT(MeSH)_2_@Si composite, which are ascribed to the doping agent of FeCl_4_^−^ [37]. For the pattern of PProDOT(MeSH)_2_@Si composite, aside from the diffraction peaks of PProDOT(MeSH)_2_, characteristic diffraction peaks of Si are observed at 28.5°, 47.4°, and 56.3°. It is clear that the intensity of the diffraction peaks of porous silicon spheres (Si) in the PProDOT(MeSH)_2_@Si composite is much lower than pure porous silicon spheres. The possible reason for this phenomenon is that the silicon spheres (Si) are uniformly coated by PProDOT(MeSH)_2_, and there is some interaction between the PProDOT(MeSH)_2_ and Si, which can lead a decrease in peak intensity of silicon spheres (Si), suggesting that the composite is not a simple physically mixing of PProDOT(MeSH)_2_ and Si.

### 3.2. Morphology Analysis

Figure 3 shows the SEM images of the SiO_2_ spheres, porous silicon spheres (Si), PProDOT, PProDOT –(MeSH)_2_ and PProDOT(MeSH)_2_@Si composite. As shown in Figure 3a, SiO_2_ possess uniform spherical morphology with an average diameter of ~360 nm and are relatively uniform and highly monodispersed. It is clear that there is no size change of the SiO_2_ sphere during the conversion from SiO_2_ to porous silicon spheres (Si) via magnesium reduction reaction. Compared with SiO_2_ spheres, the surface of porous silicon (Si) is rough (Figure 3b). In the case of PProDOT(MeSH)_2_, clumpy and rod-like structures are observed (Figure 3c). After combination with PProDOT(MeSH)_2_, the surface roughness occurring in the silicon spheres (Si) disappears, and the surface of the porous silicon spheres (Si) become smoother than before, implying the wrapping of PProDOT(MeSH)_2_ on the surface of silicon spheres (Si). Furthermore, the existence of a rod-like form in the composite can be assigned by the presence of PProDOT(MeSH)_2_.

**Figure 4:**
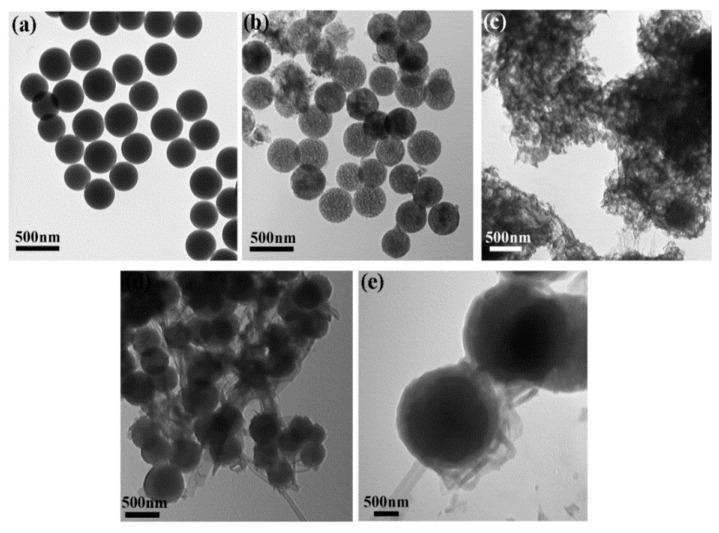
TEM images of (**a**) SiO_2_, (**b**) porous silicon sphere (Si), (**c**) PProDOT(MeSH)_2_, (**d**) and (**e**) PProDOT(MeSH)_2_@Si.

Figure 4 shows the TEM images of the SiO_2_ spheres, porous silicon spheres (Si), PProDOT –(MeSH)_2_, and PProDOT(MeSH)_2_@Si composite. Figure 4a,b shows the TEM image of SiO_2_ spheres and porous silicon spheres (Si). It is obvious that the solid SiO_2_ spheres transform into porous structures by a magnesium reduction reaction, which can be understood by the presence of bright points inside the porous silicon spheres (Si) (Figure 4b). In addition, the existence of a small number of nanostructured particles indicates that the occurrence of damage or cracking of the spherical shape of some SiO_2_ spheres in the reduction process. However, most of the porous silicon spheres (Si) are uniformly distributed and free from damage or cracking. The pure PProDOT(MeSH)_2_ displays a net-like structure (Figure 4c) constructed from the combination of the rod-like polymer matrix. The rod-like polymer matrix is clearly observed in the case of PProDOT(MeSH)_2_@Si composite (Figure 4d,e), and the porous silicon spheres (Si) become solid structure after coated by PProDOT(MeSH)_2_.

**Figure 5:**
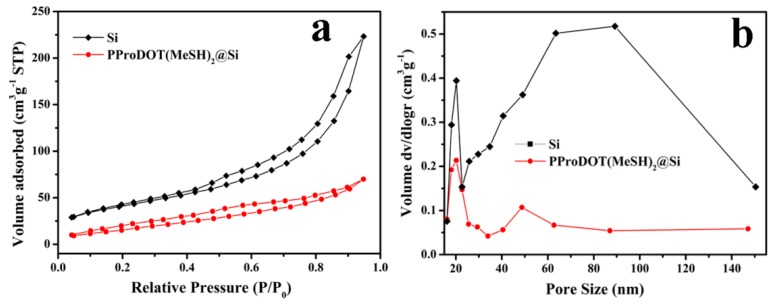
Nitrogen adsorption–desorption isotherms of (**a**) Si and PProDOT(MeSH)_2_@Si composite and (**b**) the corresponding pore radius distribution.

To probe the exact porosities of the different products, the porous silicon spheres (Si) and PProDOT(MeSH)_2_@Si composite are characterized by Brunauer−Emmett−Teller (BET) nitrogen (N_2_) adsorption–desorption measurements (Figure 5a). The BET surface areas of porous silicon spheres (Si) and PProDOT(MeSH)_2_@Si composite are calculated to be 149 and 65.7 m^2^·g^−1^, respectively. Comparing with porous silicon spheres (Si), the decrease in BET surface areas of PProDOT(MeSH)_2_@Si composite results from the coating of PProDOT(MeSH)_2_ on the surface of porous silicon spheres (Si). As shown in Figure 5b, the pore-size distribution curves of the porous silicon spheres (Si) and PProDOT(MeSH)_2_@Si are plotted based on the Barrett–Joyner–Halenda (BJH) method. At the high relative pressure region, the isotherm curves with a hysteresis loop can be seen in both samples, especially for porous silicon, which is a characteristic process between adsorption and desorption of mesopores and macropores. The total pore volume of Si and PProDOT(MeSH)_2_@Si composite is calculated to be 0.33 and 0.1 cm^3^·g^−1^, respectively. The obvious change of total pore volume suggests that the PProDOT(MeSH)_2_ distributes not only on the surface but also in the interior of porous silicon spheres (Si), which is well in accordance with the results of structural and morphological analysis, further proving that the composite is not a simple physically mixing of PProDOT(MeSH)_2_ and Si. Moreover, the low total pore volume in the case of PProDOT(MeSH)_2_@Si composite can decrease the simple adsorption of heavy metal ions caused by the porous structure of silicon spheres. As a result, this can enhance the synergetic effects between PProDOT(MeSH)_2_ and Si, and the mesoporous and microporous channels in PProDOT(MeSH)_2_@Si composite can also improve the adsorption of heavy metal ions.

### 3.3. Electrochemical Determination of Cd(II), Pb(II), and Hg(II)

**Figure 6:**
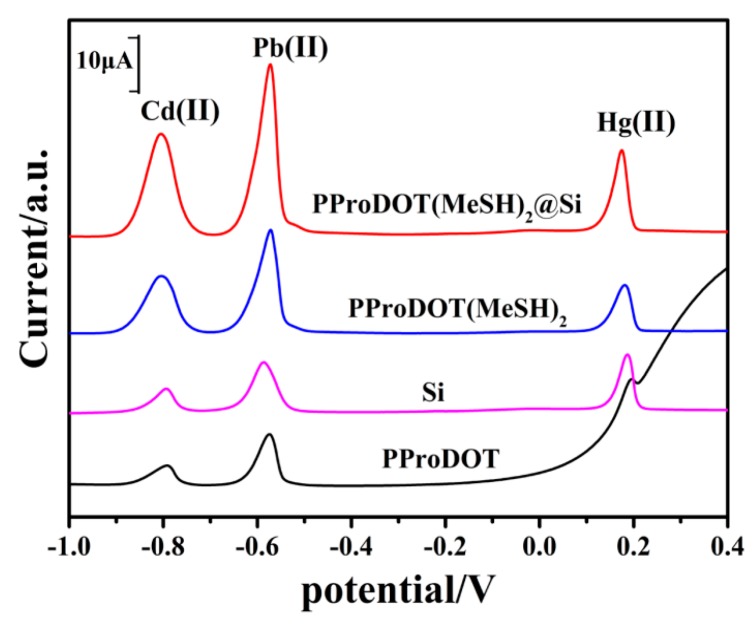
Differential pulse voltammetry (DPV) response curves of different modified glassy carbon electrode (GCE) in 0.1 M ABS (pH = 4.5) containing 1.0 μM of Pb(II), Cd(II) and Hg(II) (deposition potential: −1.4V, deposition time: 180 s, amplitude: 0.05 V, increment potential: 0.002 V, pulse width: 0.05 s, pulse period: 0.1s).

To evaluate the catalytic efficiency of different modified electrodes, porous silicon spheres (Si), PProDOT, PProDOT(MeSH)_2_, and PProDOT(MeSH)_2_@Si modified electrodes were selected to carry out the simultaneous detection of Cd^2+^, Pb^2+^, and Hg^2+^ ions by the DPV method. As shown in Figure 6, the characteristic peaks of Cd(II), Pb(II), and Hg(II) appear at potential values of about −0.8, −0.58, and −0.18V, respectively. The relative peak potentials is the same as reported for Cd(II), Pb(II), and Hg(II) [39]. It is obvious from Figure 6 that PProDOT(MeSH)_2_@Si/GCE has higher peak currents than other modified electrodes, indicating higher catalytic efficiency of the composite than other materials. It should noted that, in the case of detection of Hg^2+^ ions, PProDOT is not supposed to be ideal electrode material causing by its lower sensitivity. However, this defect is prevented after the entry of two thiol-based chains to the monomer unit, which result in an increase in sensitivity in PProDOT(MeSH)_2_. As a result, the combination of PProDOT(MeSH)_2_ and Si can improve the catalytic efficiency of electrode material by the synergetic effects between PProDOT(MeSH)_2_ and Si.

### 3.4. Optimization of Experimental Condition

**Figure 7:**
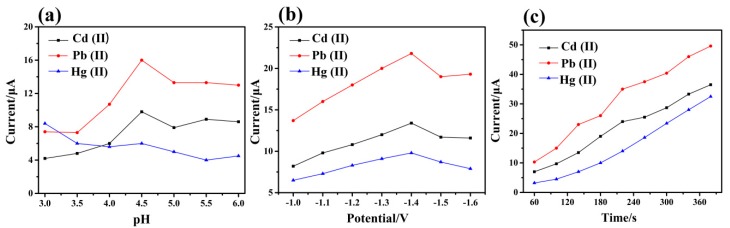
Effects of (**a**) pH values, (**b**) deposition potential (**c**) deposition time on the stripping peak currents of Cd(II), Pb(II) and Hg(II) at PProDOT(MeSH)_2_@Si/GCE (in 0.1 M ABS containing 1.0 μM of Pb(II), Cd(II) and Hg(II).

To optimize the experimental condition, simultaneous detection of Cd(II), Pb(II), and Hg(II) at the PProDOT(MeSH)_2_@Si/GCE under different pH conditions were evaluated (deposition potential, −1.4 V; deposition time, 180 s; amplitude, 0.05 V; increment potential, 0.002 V; pulse width 0.05 s; pulse period, 0.1 s). The effect of the pH value on the stripping peak currents was studied in 0.1 M ABS solution with the pH range from 3.0 to 6.0. As shown in Figure 7a, the maximum current responses of Cd(II) and Pb(II) appear at pH = 4.5. When the pH value is lower than 4.5, low DPV current responses for three target metal ions, which may be due to the protonation reaction of hydrophilic groups, can be observed. However, the peak current of Hg(II) is constantly falling, which can be resulted from the hydrolysis reaction [39]. To obtain higher sensitivity, pH value 4.5 was chosen as the optimal conditions to detect the following three target metal ions.

The deposition potential plays a significant role in the sensitivity of the modified electrode (Figure 7b). The influence of deposition potential for the response of Cd(II), Pb(II) and Hg(II) is studied. The deposition potential is varied between −1.0 and −1.6V under optimized pH conditions. It is observed that the peak currents of Cd(II), Pb(II), and Hg(II) increase remarkably from −1.0 to −1.4 V. However, the stripping response of three metal ions decreases when the potential exceeds −1.4 V, which can be attributed to the reduction of DPV measurement for the cations. As the electrolysis of water generates hydrogen bubbles at negative potentials [21], −1.4 V is chosen as the optimal deposition potential to study further. The effect of accumulation time is investigated from 60 to 400 s under ABS (pH = 4.5) conditions (Figure 7c). It is obvious that the peak currents were increased sharply in 60 to 220 s, which can be attributed to the accumulation amount of metal ions on the surface modified electrode during electrochemical deposition. When the deposition time exceeds 220 s, the increase rate of peak currents of Cd(II) and Pb(II) are not obvious, but it continuously rises for Hg(II). The reason is probably due to the working electrode–surface saturation of Cd(II) and Pb(II). Hence, the deposition time 220 s was selected for all subsequent electrochemical analysis.

### 3.5. Individual Determination of Cd(II), Pb(II) and Hg(II)

Individual detection of Cd(II), Pb(II), and Hg(II) were carried out under optimized experimental conditions using PProDOT(MeSH)_2_@Si/GCE. DPV was used as an analytical method for the electrochemical detection of Cd(II), Pb(II), and Hg(II) in 0.1M ABS (PH=4.5). The stripping peaks towards Cd(II), Pb(II), and Hg(II) appear at potentials of −0.85, −0.62, and −0.18 V, respectively. The peak current values display linearly with the increasing of Cd(II), Pb(II), and Hg(II) concentration. The related data of individual detection of three target metal ions are illustrated in Table 1.

### 3.6. Evaluation of Mutual Interferences

**Figure 8:**
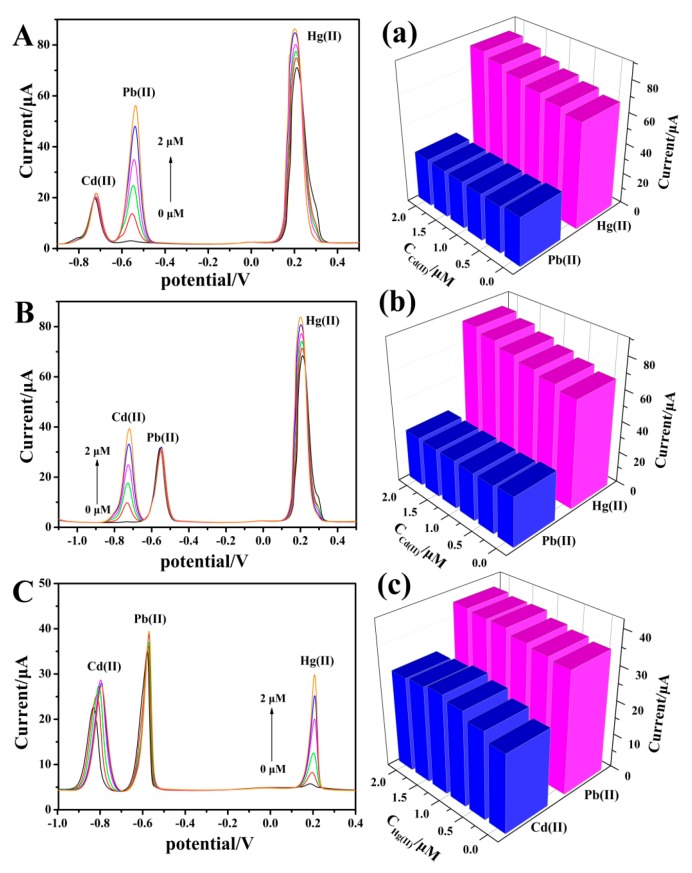
DPV response of the PProDOT(MeSH)_2_@Si/GCE at: (**A**) Pb(II) (0–2.0 μM), 1.0 μM Cd(II) and Hg(II); (**B**) Cd(II) (0–2.0 μM), 1.0 μM Pb(II) and Hg(II); (**C**) Hg(II) (0–2.0 μM), 1.0 μM Pb(II) and 1.0 μM Cd(II). (**a**), (**b**) and (**c**) comparison of the stripping peak currents of Pb(II) and Hg(II), Cd(II) corresponding to panel **A**, **B** and **C**.

To evaluate the interferences between Cd(II), Pb(II), and Hg(II) during the simultaneous detection, the following experiments were carried out. Figure 8A shows the DPV response of PProDOT(MeSH)_2_@Si/GCE at different concentrations of Cd(II) (0.0–2.0 μM) in 1.0 μM Pb(II) and 1.0 μM Hg(II). It can be observed that with the addition of Cd(II), the peak current of Hg(II) increased linearly (68.83–83.2μA) while the peak current of Pb(II) is almost unchanged (Figure 8a).

Figure 8B shows the DPV response of PProDOT(MeSH)_2_@Si/GCE at different concentrations of Pb(II) (0.0–2.0 μM) in 1.0 μM Cd(II) and 1.0 μM Hg(II). From Figure 8b, it can be seen that the peak current of Cd(II) almost remain stable with the increase of Pb(II), and the peak current of Hg(II) rose linearly from 71.5 to 86.03 μA. However, when the concentration of Cd(II) reached a certain value (1.2 μM), the peak current of Hg(II) began to stabilize. This result is probably due to the formation of a small amount Pb film and followed by the formation of Pb–Hg metallic compounds during the deposition process [39,40,41].

Figure 8 part C shows the DPV response of PProDOT(MeSH)_2_@Si/GCE at different concentrations of Hg(II) (0.0–2.0 μM) in the presence of 1.0 μM Pb(II) and 1.0 μM. It is clear that the peak current of Pb(II) changes is not obvious with the increasing of Hg(II) concentration(0 to1.2 μM), while the peak current of Cd(II) increases linearly from 23.1 to 28.3 μA and shifted slightly (Figure 8C,c). With a concentration of Hg(II) up to 1.2μM, the peak current of Pb(II) and Cd(II) remained stable, and the potential of Cd(II) was almost unchanged which may be attributed to the competition between multiple metal ions on the electrode surface. The peak current does not change after the concentration of Hg (II) reached a certain value (1.0 μM).

### 3.7. Simultaneous Determination of Cd(II), Pb(II), and Hg(II)

**Figure 9:**
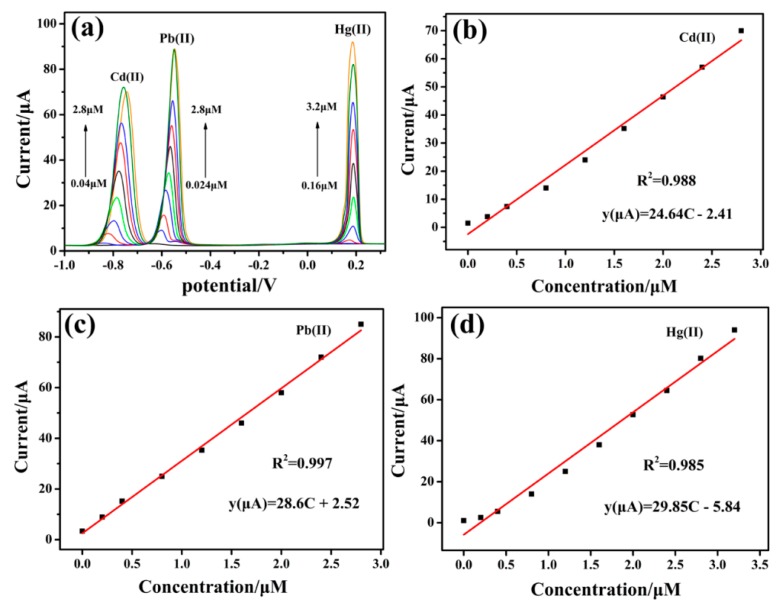
(**a**) DPV response for simultaneous analysis of the Cd(II), Pb(II), and Hg(II) at the PProDOT(MeSH)_2_@Si/GCE; (**b,c,d**) corresponding calibration curves of Cd (II) Pb(II) and Hg(II).

Under the optimal experimental conditions Cd(II), Pb(II), and Hg(II) are determined simultaneously at the PProDOT(MeSH)_2_@Si/GCE using DPV method. Figure 9a shows the DPV responses toward Cd(II), Pb(II), and Hg(II) at different concentrations. The well-separated characteristic peaks appeared at about −0.8, −0.57, and −0.18V for Cd(II), Pb(II), and Hg(II), respectively. The limit of detection is estimated according to the limit of detection LOD = 3 S/N (Table 2). The corresponding calibration curve of Cd(II) was built from 0.04 to 2.8 μM (Figure 9b). The linear equation is i/μA = −2.41 + 24.64C/μM, with the corresponding correlation coefficient of 0.988 (inset of Figure 9b). Similarly, it can be observed that corresponding calibration curves of Pb(II) and Hg(II) were built at 0.024 to 2.8 μM and 0.16 to 3.2 μM, and the corresponding regression equations are i/μA = −2.41 + i/μA = 2.52 + 28.6C/μM and i/μA= −5.84 + 29.85C/μM (Figure 9c,d). The corresponding correlation coefficient of 0.997 and 0.985 for Pb(II) and Hg(II) (inset of Figure 9c,d), respectively. The linear range and detection limit toward Cd(II), Pb(II), and Hg(II) are compared with other reports using different electrode sensor materials as illustrated in Table 2.

**Figure 10:**
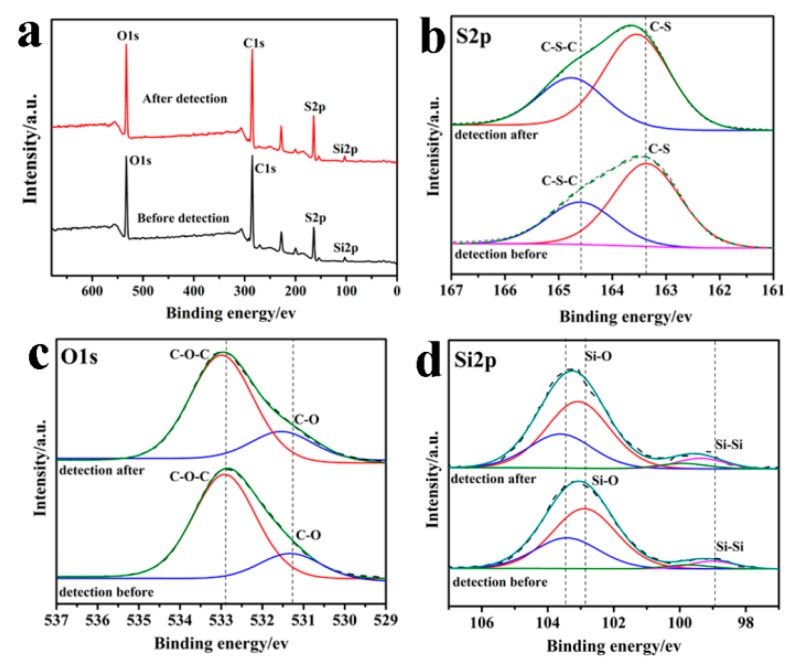
X-ray photoelectron spectroscopy (XPS) spectrum of: (**a**) PProDOT(MeSH)_2_@Si before and after detection of Cd(II), Pb(II), and Hg(II); high-resolution scans of (**b**) S2p, (**c**) O1s, and (**d**) Si2p.

The chemical state for each element of PProDOT(MeSH)_2_@Si was investigated by X-ray photoelectron spectroscopy (XPS) before and after detection. As displayed in Figure 10a, the four peaks at 533.08, 285.08, 164.08, and 103.8 eV are detected in the XPS survey spectrum of PProDOT(MeSH)_2_@Si, which corresponds to O1s, C1s, S2p, and Si2p, respectively. These results indicate the presence of O, C, S, and Si atoms on the PProDOT(MeSH)_2_@Si.

Figure 10b displays high resolution XPS spectrum of S2p peaks for PProDOT(MeSH)_2_@Si before and after detection of three target metal ions. It is clear that the two binding energy of C–S (163.38, 164.58 eV) shifted to the high binding energy region (163.58, 164.78 eV), respectively, which suggested that there is a complex between positively charged metal ions and negatively charged functional group (–SH). Figure 10c shows the high resolution O 1s peaks of PProDOT(MeSH)_2_@Si. Before the detection of target metal ions, the peak of C–O and C–O–C appears at 531.28 and 532.88 eV. It can be observed that the binding energy of C–O and C–O–C shifted to 531.58 and 532.98 eV after detection [42]. According to the high resolution XPS spectrum Si2p peaks of PProDOT(MeSH)_2_@Si (Figure 10d), the binding energy of Si–Si at 98.91 eV slightly shift to the high energy region (99.38 eV). This result suggests that the possible interaction between Si and metal ions. Moreover, Si2p peaks also show the two peaks at 102.85 and 103.4 eV, which are attributed to the Si–O band [43]. The binding energy of Si–O shifted from 102.85 and 103.4 eV to 103.09 and 103.68 eV, respectively. Which suggests that the heavy metal ions were adsorbed by the porous silicon in the PProDOT(MeSH)_2_@Si composite. On the basis of the results above, it can be concluded that the element of S and Si in the PProDOT(MeSH)_2_@Si composite contribute to the adsorption of target metal ions during the deposition step.

### 3.8. Reproducibility and Stability of Modified Electrode Study

**Figure 11:**
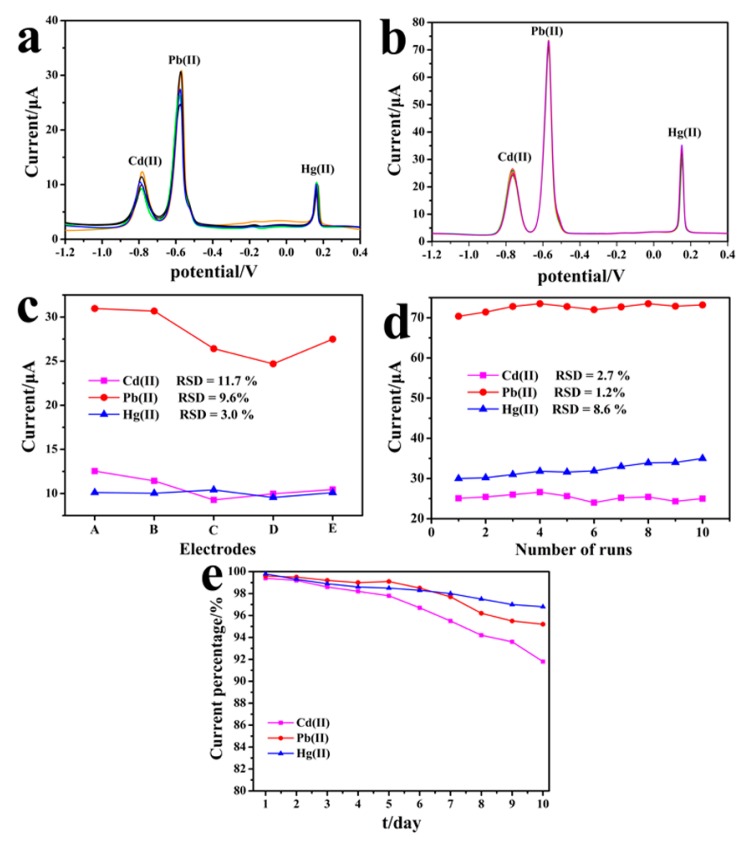
DPV response of 1.0 µM Cd(II), Pb(II), and Hg(II) at PProDOT(MeSH)_2_@Si/GCE for the (**a**) reproducibility and (**b**) repeatability. The peak current value change (**c**) and (**d**) corresponding to (**a**) and (**b**), (**e**) Stability curves PProDOT(MeSH)_2_@Si/GCE. RSD refers to the relative standard deviation.

To further evaluate the sensing performance of PProDOT(MeSH)_2_@Si/GCE, reproducibility and repeatability experiments were carried out in 0.1 M ABS solution (pH = 4.5) containing 1.0 μM Cd(II), Pb(II), and Hg(II) under the optimized conditions. The reproducibility of modified electrode was studied with five different PProDOT(MeSH)_2_@Si/GCEs which were prepared independently by the same procedure. As shown in Figure 11a, the peak currents of Cd(II) and Pb(II) are slightly changed, and the relative standard deviations (RSDs) of five parallel experiments determined to be 11.7%, 9.6%, and 3.0% for Cd(II), Pb(II), and Hg(II), respectively (Figure 11c), which also demonstrates the reliability of the fabrication procedure. At the same condition, the repeatability of the PProDOT(MeSH)_2_@Si/GCE was carried out at same electrode for ten runs (Figure 11b). As shown in Figure 11b, there are not any alterations for the peak currents of three metal ions, and the RSDs were calculated to be 2.7%, 1.2%, and 8.6% for Cd(II), Pb(II), and Hg(II) (Figure 11d), respectively. In addition, the stability of PProDOT(MeSH)_2_@Si/GCE is observed by applying the same modified electrode stores at room temperature and tests every day. As shown in Figure 11e, the current response change of the PProDOT(MeSH)_2_@Si/GCE is 91.8%, 95.2%, and 96.8% for Cd(II), Pb(II), and Hg(II) of the initial value after 10 days. The above results indicate that the PProDOT(MeSH)_2_@Si/GCE modified electrode has quite good reproducibility, repeatability, and stability.

### 3.9. Real Sample Analysis

PProDOT(MeSH)_2_@Si/GCE for simultaneous determination of Cd(II), Pb(II), and Hg(II) showed high sensitivity and better reproducibility. To evaluate the practical application of the PProDOT(MeSH)_2_@Si/GCE, tap water samples analysis were performed under the best experimental conditions. First, the tap water samples were acidified to pH = 4.5 with HAc and NaAc. Subsequently, standard solutions of Cd(II), Pb(II) and Hg(II) with different concentrations were added to the tap water samples. The recovery results are listed in Table 3, and it can be seen that the recoveries of the Cd(II), Pb(II), and Hg(II) are 92%–104%, 93.3%–105%, and 97.0%–106%, respectively. Therefore, it could be concluded that the proposed electrode could be used for detection of Cd(II), Pb(II), and Hg(II) in tap water samples.

## 4. Conclusions

In this paper, we presented an electrochemical sensor based on composite material (PProDOT(MeSH)_2_@Si) from the incorporation of thiol(–SH) grafted PProDOT with porous silicon spheres (Si) for the electrochemical detection of heavy metal ions (Cd(II), Pb(II), and Hg(II)). It was found that the PProDOT(MeSH)_2_ can incorporate with porous silicon spheres, and the PProDOT(MeSH)_2_ distributed not only on the surface but also in the interior of porous silicon spheres (Si), indicating that the composite was not a simple physically mixing of PProDOT(MeSH)_2_ and Si. This kind of combination structure between PProDOT(MeSH)_2_ and porous silicon spheres (Si) could be a benefit for enhancing the interactions between PProDOT(MeSH)_2_ and porous silicon spheres (Si) that can improve the synergetic effects. Furthermore, the mesoporous and microporous channels in PProDOT(MeSH)_2_@Si composite could also improve the adsorption of heavy metal ions. The results from electrochemical performance showed that the entry of two thiol-based chains to monomer unit resulted in an increase of sensitivity in PProDOT(MeSH)_2,_ which was related to the interaction between thiol group (–SH) and heavy metal ions. And, the combination of PProDOT(MeSH)_2_ with Si can improve catalytic efficiency electrode material. As a result, the PProDOT(MeSH)_2_@Si/GCE exhibited high selectivity and sensitivity, and possessed a low mutual interference with high selectivity for simultaneous detection of Cd(II), Pb(II), and Hg(II) ions, demonstrating potential application in the field of electrochemical sensor for detection of heavy metal ions.

## Figures and Tables

**Table 1:** Performance of the PProDOT(MeSH)_2_@Si composite-modified glassy carbon electrode (GCE) for individual determination of Cd(II), Pb(II) and Hg(II).

Analyties		Cd(Ⅱ)		Pb(Ⅱ)		Hg(Ⅱ)	
	**N.D**	(ìM)	(ìA)	(ìM)	(ìA)	(ìM)	(ìA)
	1	0.04	2.4	0.024	3	0.07	3
	2	0.2	5	0.3	10	0.4	6
	3	0.4	7.3	0.8	20	0.8	10
	4	0.8	12	1.2	29	1.6	17
	5	1.2	23.5	2	44	2.8	24.5
	6	1.6	29.2	2.4	50	3.6	29
	7	1.8	34	2.8	55	4.8	36
	8	2.2	44.5	3.2	60	5.6	40.2
	9	2.6	48.5	3.6	65	6.4	44
	10	3	53	4	70	7.6	50
Liner range		0.04–3.0		0.024–4.0		0.07–7.6	
Regression Equation		i(μA) = −0.457 + 17.08C(μM)		i(μA) = −0.457 + 17.08C(μM)		i(μA) = −0.457 + 17.08C(μM)	
R^2^		0.983		0.983		0.98	
LOD(nm)		8.3		4.7		8.0	

N.D: Numbers of detection.

**Table 2:** Comparison of different sensors for the determination of heavy metals.

Electrode	Methods	Analytes	Linear Range (µM)	LOD (nM)	Ref.
Nafion-HAP	DPASV	Cd^2+^ Pb^2+^ Hg^2+^	0.1–10 0.1–10 0.1–1.0, 3.0–10.0	35 49 30	[44]
[Ru(bpy)_3_]^2+^-GO/Au	DPSV	Cd^2+^ Pb^2+^ Hg^2+^	0.02–0.9 0.05–1.5 0.1–0.9	2.8 1.14 1.6	[45]
rGO-Fe_3_O_4_	SWASV	Cd^2+^ Pb^2+^ Hg^2+^	0.1–1.7 0.1–1.7 0.1–1.7	28 8 17	[41]
poly(BPE)HNs	DPV	Pb^2+^	0.012–4	5	[20]
MgFe-LDH/graphene	SWASV	Cd^2+^ Pb^2+^	0.1–1.0 0.1–1.0	5.9 2.7	[46]
N-doped graphene	DPSV	Cd^2+^ Pb^2+^ Hg^2+^	0.05–9.0 0.01–9.0 0.2–9.0	50 5 50	[47]
poly(BPE)/g-C_3_N_4_	DPV	Cd^2+^ Pb^2+^	0.12–7.2 0.08–7.2	18 3.34	[21]
Grapheme-CeO_2_	DPASV	Cd^2+^ Pb^2+^ Hg^2+^	0.2–2.5 0.2–2.5 0.2–2.5	0.1994 0.1057 0.2771	[40]
N-HCS/CS/gold	SWASW	Pb^2+^ Hg^2+^	0.05–0.7 0.01–0.9	15 2.35	[48]
PProDOT(MeSH)_2_@ Si	DPV	Cd(Ⅱ) Pb(Ⅱ) Hg(Ⅱ)	0.04–2.8 0.024–2.8 0.16–3.2	5.75 2.7 1.7	this work

**Table 3:** Determination of Cd(II), Pb(II) and Hg(II) in tap water.

Original (µM)	Added (µM)			Found (µM)			Recovery (%)		
	Cd(II)	Pb(II)	Hg(II)	Cd(II)	Pb(II)	Hg(II)	Cd(II)	Pb(II)	Hg(II)
**N.D**	0.5	0.5	0.5	0.52	0.53	0.49	104	105	97.2
**N.D**	1.0	1.0	1.0	0.92	1.01	0.97	92.0	101	97.0
**N.D**	1.5	1.5	1.5	1.45	1.4	1.59	96.6	93.3	106
**N.D**	2.0	2.0	2.0	2.06	2.06	1.94	103	103	97.2

N.D: not detected.
